# Sheep Brucellosis in Kuwait: A Large-Scale Serosurvey, Identification of *Brucella* Species and Zoonotic Significance

**DOI:** 10.3390/vetsci7030132

**Published:** 2020-09-08

**Authors:** Yousef Al-Sherida, Adel H. El-Gohary, Amro Mohamed, Mohamed El-Diasty, Gamal Wareth, Heinrich Neubauer, Adel Abdelkhalek

**Affiliations:** 1 Animal Health Department, Division of Epidemiology and Zoonoses, Public Authority of Agriculture Affairs & Fish Resources (PAAFR), Kuwait City 13075, Kuwait; asraa55@hotmail.com; 2 Department of Hygiene and Zoonoses, Faculty of Veterinary Medicine, Mansoura University, Mansoura 35516, Egypt; adelelgohary@yahoo.com (A.H.E.-G.); kombanymeras@yahoo.com (A.M.); 3 Animal Health Research Institute, Mansoura Provincial Lab, Mansoura 35516, Egypt; dr_mesbah_m@yahoo.com; 4 Friedrich-Loeffler-Institut, Institute of Bacterial Infections and Zoonoses (IBIZ), Naumburger Str. 96a, 07743 Jena, Germany; heinrich.neubauer@fli.de; 5 Faculty of Veterinary Medicine, Benha University, Moshtohor, Toukh 13736, Egypt; 6 Department of Food Hygiene and Control, Faculty of Veterinary Medicine, Mansoura University, Mansoura 35516, Egypt; abdelkhaleka@hotmail.com

**Keywords:** ovine brucellosis, seroprevalence, Kuwait, *Brucella melitensis*, zoonosis

## Abstract

Brucellosis is a common zoonotic disease of major concern in humans of Kuwait, and *B. melitensis* causes most human cases. The disease is endemic in small ruminants, cattle, and camels for decades, causing substantial economic losses in livestock production. However, a nationwide large-scale investigation of brucellosis in the small ruminant population has not been done in the past two decades. A serosurvey of sheep brucellosis in the five districts of Kuwait with most animal production farms was done between 2016 and 2019. In total, 67,054 serum samples from 233 sheep herds were collected and tested. Additionally, milk and tissue samples were collected from 46 seropositive cases for bacteriology. Thirty persons from seven seropositive farms were tested by serology. The incidence of seropositive cases was 7% in districts devoid of vaccination, while it was 4.7% in farms with history of vaccination. The serosurvey revealed that 89% of non-vaccinated herds (*n* = 181) were seropositive by Rose Bengal test (RBT), buffered acidified plate antigen test (BAPAT), and complement fixation test (CFT). Prevalence of 100% was reported for non-vaccinated sheep herds from Al-Wafrah and Al-Jahra districts, followed by those from Al-Salmi (88.24%), Al-Abdali (86.7%) and Kabd (75.6%). Implementation of vaccination with *B. melitensis* Rev.1 vaccine and test-and-slaughters in 20 herds reduced the seroprevalence to 33.3% and 25% in herds from Al-Jahra and AL-Wafrah, respectively. *B. melitensis* was isolated from 20 samples (43.5%). More than half of the examined animal owners (56.6%) tested positive for *Brucella* using RBT, BAPAT and CFT. The high numbers of infected herds and high prevalence in herdsmen are alarming. Thus, control measures have to be ensured immediately. The epidemiological situation in Kuwait is similar to those of the neighboring countries and the combined action of these states is needed. The understanding of the economic and public health impact of brucellosis in Kuwait needs to grow.

## 1. Introduction

Brucellosis is one of the most frequently encountered bacterial zoonosis globally. The disease affects domesticated animals and wildlife as well as humans, causing substantial economic losses in the livestock industry due to abortion, reproductive failure, sterility and drops in milk production, and significant public health problems [1,2]. Despite the disease being notorious in veterinary medicine, its importance, diagnosis, and control have attracted little attention in human medicine. The World Health Organization (WHO) considered brucellosis a neglected disease [3]. The pathogen is crossing host barriers and spreading to unusual hosts as well [4]. The disease is endemic in the Middle East, Mediterranean countries, and the Arabian Gulf area among humans and animals, and prevalences in small ruminant populations are among the highest worldwide [5]. *Brucella* (*B.*) *melitensis* is the primary cause of brucellosis in sheep and goats. Sheep and goats are also significant reservoirs for maintenance, spread and transmission. Due to grazing regimes, ways of rearing and management systems in place, these animals maintain the infection and shed pathogenic agents into the environment [6]. The course of the disease and clinical picture in certain breeds of sheep is similar to that in goats. *Brucella melitensis* is also the member of the genus *Brucella* with the highest zoonotic risk, and most human cases are caused by *B. melitensis* worldwide. Although many developing countries in the Middle East have implemented highly restrictive control programs, the disease is still endemic, resulting in significant public health problems in most of the Middle Eastern countries [5,7].

Kuwait is a small desert country located in the northwestern corner of the Arabian Gulf, with around 4.2 million inhabitants in 2020. The sheep population in the country was 920,613 animals in 2017 with a density of 51.66 (units per square kilometer) according to the World Organisation for Animal Health (OIE), in 2017 [8]. Brucellosis is a disease of significant concern in humans [9,10,11,12,13]. *Brucella melitensis* strains that were isolated from Mubarak Al-Kabeer, Farwaniya and Amiri Hospitals of Kuwait showed a close relationship to UAE and East Mediterranean strains [11]. In 2006, the department of public health and infectious diseases of the Kuwaiti Ministry of Health finally declared brucellosis an endemic disease. The disease has been prevalent in cattle, sheep and goats, and camels since the latter part of the last century [14,15], with a seroprevalence of 5.2%, 6.6% and 7.7%, respectively, in 1989. *Brucella melitensis* was isolated from cows, sheep, and goats. In 1993, studies of a flock showed that 9.4% of sheep were seropositive. In 1994, the seroprevalence had increased even up to 14%. A nationwide survey of sheep revealed a prevalence of 2.18% in 1997 [16]. The nomadic lifestyle of the country made sheep meat and milk an essential part of the daily local diet. However, data and information on current prevalence and circulating biotypes of *Brucella* are missing. Therefore, a large-scale serosurvey in sheep herds in Kuwait as well as isolation and identification of brucellae was done. As brucellosis is considered an occupational disease of breeders of small ruminants, a study of herd owners was initiated to gain preliminary data for the risk assessment.

## 2. Materials and Methods

### 2.1. Study Area and Animal Population

**Figure 1:**
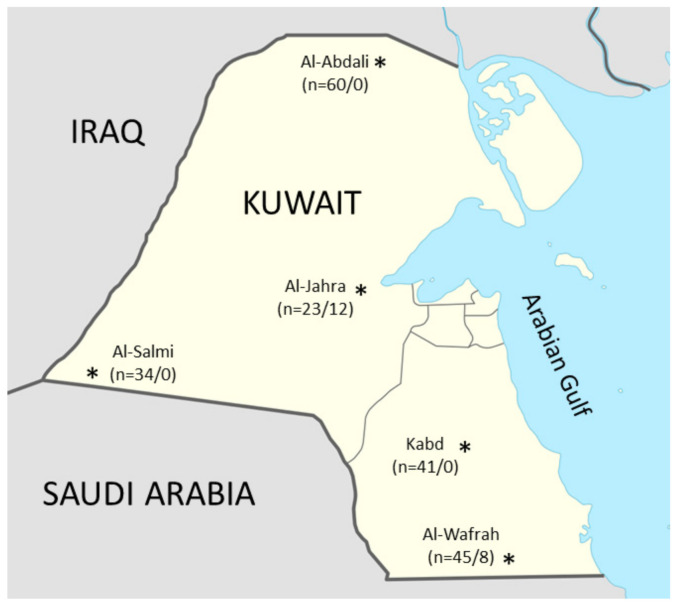
Map of the state of Kuwait showing the five districts holding the majority of sheep herds in the country, and *n* (non-vaccinated/vaccinated) is the number of tested herds.

A large-scale serosurvey of brucellosis in sheep was conducted from 2016 to 2019 in five different districts of Kuwait (Figure 1). AL-Wafrah is the southernmost area of Kuwait at the Saudi Arabian border. Al-Abdali is the northernmost area of Kuwait at the Iraqi border. Al-Wafrah in the south and Al-Abdali in the North are the only two cities of Kuwait with farming and animal keeping. The third district is Al-Salmi with borders to Kuwait and Saudi Arabia. Kabd and Al-Jahra districts are two districts located in the center of Kuwait. These five regions are well-known places for their highly organized sheep farms. All semi-large farms of these districts, with a density of over than 100 sheep, take part in the national program on sheep brucellosis. This study included 223 sheep herds with a total number of 67,054 animals. The total number of tested animals represents around 7% of the total sheep population in Kuwait. Small herds with a population of less than 100 sheep were not included in the survey. Most of the investigated herds are migratory flocks. A total of 203 assumed healthy non-vaccinated herds (60 herds from Al-Abdali, 45 herds from AL-Wafrah, 41 herds from Kabd, 34 herds from Al-Salmi, and 23 herds from Al-Jahra), and 20 herds (12 herds from Al-Jahra and eight from Al-Wafrah) with a history of vaccination with *B. melitensis* Revi.1 vaccine were sampled (Table 1). Herds were vaccinated during a test-and-slaughter program of the veterinary authorities of Kuwait. Samples have been collected from vaccinated herds one year after vaccination to avoid the potential cross-reactions produced by antibodies induced by the vaccine. All animals (100%) in the selected visited herds were sampled and examined.

### 2.2. Sample Collection

All sheep on the visited farms were sampled. An amount of 5 mL of blood was collected from the jugular vein of each animal and centrifuged. Sera were collected immediately in Eppendorf tubes and kept in the fridge at 4 °C degree until use. In addition to the blood samples, thirty milk samples were collected from non-vaccinated seropositive ewes. Liver, spleen, and lymph node samples were collected from eight non-vaccinated seropositive ewes after slaughtering. The eight ewes had been subject to accidental slaughtering during sample collection. Stomach content, spleen, liver, and lung specimens were taken from eight aborted foeti of non-vaccinated ewes that had been found unexpectedly in the visited farms before disposal. All samples were collected in sterile plastic bags and sent to the Public Authority for Agriculture Affairs and Fish Resources (PAAF) Kuwait City, Kuwait for bacterial isolation and identification.

**Human samples:** Only thirty sheep holders from seven seropositive farms agreed to give blood samples for serology. Eleven persons were working in three farms in Al-Wafra, eight were working in two farms in Al-Salmi and eleven persons were working in two farms in Al-Jahra. The donors had no contact with the *B. melitensis* Rev1 vaccine, which is infectious for humans. Before sampling, written and oral consent was taken from each sheep holder. Approval was obtained from the ethical committee at the office of the Deputy Director General (DDG) for the Animal Wealth sector in the PAAF.

### 2.3. Serological Tests

All sheep and human sera were tested by the Rose Bengal test (RBT) and buffered acidified plate antigen test (BAPAT) as screening tests. Positive serum samples in both the RBT and BAPAT were confirmed using the complement fixation test (CFT) as previously described [17]. Sheep and human sera that gave positive reactions in the RBT, BAPAT and CFT were considered positive for brucellosis. To avoid the potential cross-reaction with the antibodies released from vaccination, samples from vaccinated herds were collected 12–18 months post-vaccination, and were considered positive after confirmation by the RBT, BAPAT and CFT. Antigens and tests used for RBT and BAPAT were obtained from the Veterinary Serum and Vaccine Research Institute, Abbassia, Cairo, Egypt while antigen and materials for CFT were offered by the National Veterinary Services Laboratories (NVSL), Ames, IA 50010, USA. All reagents used for the serology have been standardized in accordance with the OIE Manual.

### 2.4. Bacteriological Isolation and Identification

Isolation was carried out from milk samples, samples of aborted foeti and tissue specimens. *Brucella* identification and biotyping were carried out according to Alton et al. [17]. Colony morphology, urease, oxidase, catalase, CO_2_ requirement, H_2_S production, growth in the presence of thionin and fuchsine dyes, agglutination with mono-specific anti-sera (A, M, R), reaction with trypaflavine and crystal-violet were investigated. According to the manufacturer’s instructions, DNA was extracted from heat-inactivated biomass using the High-Pure template preparation kit (Roche Applied Sciences, Mannheim, Germany). DNA content was measured, and *Brucella* species were molecularly confirmed by (Abortus, Melitensis, Ovis, Suis) AMOS-PCR [18]. All isolates have been identified at the species level and biovars have not considered. Culturing has not been applied on human samples.

## 3. Results

A nationwide survey of sheep brucellosis in Kuwait with RBT, BAPAT, and CFT revealed the presence of brucellosis in 89% of tested non-vaccinated sheep herds. In total, 181 herds of the five regions investigated were positive in serology with a total animal prevalence of 7% (Table 2). The highest herd prevalences were detected in Al-Wafra and Al-Jahra districts and reached 100% in non-vaccinated herds, followed by Al-Salmi, Al-Abdali and Kabd districts with herd prevalences of up to 88.24%, 86.7%, and 75.6%, respectively. The lowest herd prevalences were detected in the vaccinated herds at Al-Wafra and Al-Jahra with herd prevalences of 25% and 33.3%, respectively. Al-Abdali and AL-Wafrah, both known for farming and animal production, had animal prevalences of 9% and 6.5%, respectively. Al-Jahra showed the highest animal prevalence of 10.8%, while Kabd showed the lowest prevalence of 4.7% (Table 2).

Forty-six samples were collected from aborted fetuses, as well as tissue specimens and milk of seropositive ewes for bacteriology. *Brucella* was isolated from 20 out of 46 milk and tissue samples with an isolation rate of 43.5%. All samples collected from aborted foeti gave positive culture (*n* = 8), while only 33% (*n* = 10) and 25% (*n* = 2) of samples collected from milk and tissues of slaughtered seropositive ewes were culture positive, respectively (Table 3). All *Brucella* strains have been identified as *B. melitensis* by classical bacteriology. AMOS-PCR confirmed *B. melitensis*.

Investigation by serology of brucellosis among sheep holders revealed that 56.6% of tested persons were seropositive with RBT, BAPAT, and CFT. Only 30 persons (owners and workers) from seven positive farms provided samples and at least one person was positive at each farm. All sheep holders (*n* = 3) at one farm from Al-Salmi district were seropositive, and six from eight examined staff at another farm in Al-Jahra district were positive for brucellosis (Table 4). Culturing has not been done on human samples.

## 4. Discussion

In Kuwait, brucellosis is one of the most critical endemic zoonoses [5,16]. The disease has been prevalent in humans [10,11] and the livestock population [14,15] for a long time. However, a recent nationwide survey of brucellosis in the animal population is missing. The main objective of this study was to gain preliminary data on brucellosis prevalence in the sheep population reared in the main five districts of sheep farming in Kuwait to better understand the epidemiological situation and to give advice for better management if necessary.

Districts Al-Wafrah and Al-Salmi showed 100% and 88.24% prevalences in the tested sheep herds, respectively. Both districts are located in the southern part of the country and are the port borders of Kuwait–Saudi Arabia, followed by Al-Abdali in the North with a herd prevalence of 86.7%. Al-Abdali is located at the northern border of Kuwait and parallels with the Kuwait–Iraq border. Kuwait shares extensive, however, loose borders with neighboring Iraq and Saudi Arabia. Both countries are endemic for brucellosis and control of sheep brucellosis in Iraq and Saudi Arabia is very challenging due to the nomadic animal production system of those countries [5,7,16]. Illegal transboundary transportation of diseased animals is unlikely to occur because of effective border control. However, trade of non-symptomatic carrier hosts and wildlife might play a role in transboundary spreading. Testing for brucellosis at the borders is uncommon. Although the disease is endemic in the three countries, little research has been conducted on sheep brucellosis. In this setting, the development of control strategies for brucellosis requires coordinated regional control efforts of the neighboring countries. In addition to the three border districts, two centrally located areas were selected for screening to include the five farming and animal production districts of Kuwait and capture the majority of the sheep population. Extensive animal farming and nomadic husbandry are potential risk factors for a high prevalence of brucellosis in sheep. Indeed, all investigated herds are mobile herds grazing in large-scale areas. However, they are well controlled by the concerned authorities. Collecting data on the number of animals and health status has begun and they are subjected to the national survey on sheep brucellosis, which started in 2016. This program includes test-and-slaughter strategy and mass vaccination of all animals with the *B. melitensis* Rev.1 vaccine. Test-and-slaughter has been applied to all positive herds, but vaccination is not practiced for all herds yet. The screening was carried out in the semi-large farms with a density of over 100 sheep, representing only 7% of the total sheep population in Kuwait. Nevertheless, investigation of brucellosis in small herds at small sheep breeders has to be taken into consideration.

All 68 tested non-vaccinated herds were seropositive in Al-Jahra and Al-Wafrah, while of the 20 vaccinated herds, 33.3% and 25% in the same districts were seropositive, respectively. *B. melitensis* Rev.1 vaccine is the best choice for the control of brucellosis in sheep and recommended by the OIE; however, its use subcutaneously creates serological immune responses that interfere with the serological diagnosis [19]. To avoid the potential cross-reaction with the antibodies released from immunization, the vaccinated herds have been tested at least after one-year post-vaccination and some herds were sampled 18–24 months post-vaccination. Moreover, CFT has been applied as a confirmatory test that can distinguish the infection cases successfully. Kuwait is an endemic country of brucellosis and this work has been applied as an emergency plan to eliminate brucellosis from sheep herds in the country. Isolation is still the gold standard for diagnosis but in the endemic countries where brucellosis is present in 100% of herds in some districts, an emergency plan is required to eradicate the disease. Thus, three different serological tests have been applied. All seropositive cases in the three used serological tests were considered *Brucella* positive cases and eliminated from the herds. Samples were collected from vaccinated farms one year after vaccination to assess the efficacy of the vaccination program on the prevalence of ovine brucellosis. It has to be noted that the vaccinated herds were tested positive with smooth LPS (*B. abortus*)-based tests in this study. Indeed, after vaccination of 350,000 sexually mature sheep and goats in Kuwait with a reduced dose of *B. melitensis* strain Rev.1 in 1992, the incidence of brucellosis was lowered and the program was considered successful as the number of ovine abortions and human cases was reduced [15]. Subcutaneous mass vaccination of the small ruminant population with a reduced dose of *B. melitensis* Rev.1 strain was practiced again from 1993 to 1997 and reduced the prevalence of *B. melitensis* infection from 5.8% to 2.02% [20]. It has to be stressed that the highest prevalence ever documented in Kuwait (7%) was found in this study, underlining the failure of the control of brucellosis in the last two decades.

All isolates were diagnosed as *B. melitensis.* Sheep and goats are the natural reservoirs for *B. melitensis.* It is the dominant *Brucella* species in the Middle East and causes the majority of human cases in Kuwait [11,21]. Thus, the epidemiologic situation is coherent: there is a continuous spillover from a chronically infected sheep population to the Kuwaiti consumers. *Brucella melitensis* in sheep poses a high potential risk for human infection and is a serious public health threat. It is also a significant threat to the livestock population, livestock owners, abattoir workers, meat vendors, and professional animal health workers in Kuwait. More than half (56.6%) of sheep owners and farm workers participating in the current study were seropositive for brucellosis, a finding that fits the epidemiologic situation. Knowing that exposure to the live vaccine *B. melitensis* Rev. 1 can cause disease in humans [22], all seropositive persons in the current study were from non-vaccinated farms and had no previous contact with the *B. melitensis* Rev1 vaccine. The nomadic production system and the climate of the country are not favorable for the rearing of cattle. Thus, sheep meat and milk are still a popular and essential part of the daily diet in Kuwait. The high number of persons found infected in this study is caused by direct contact with diseased animals at non-vaccinated farms, and it can be assumed that some risky traditions such as drinking raw milk, which is famous among herdsmen in the nomadic culture, also cause forward transmission. Human brucellosis cases can serve as an indication of the prevalence of brucellosis in domestic animals. Eradication of brucellosis in animals is the key to preventing consumers and the persons involved in the value chain of sheep population from infection.

This study also highlights some of the challenges encountered during sampling. Only 30 persons from seven infected farms agreed to participate. The lack of cooperation caused difficulties in investigating a representative number of sheep owners and farm workers. Some of the farmers were unwilling to allow taking samples from their livestock because of the misconception that sampling may harm the animals or the animals may be confiscated, which would negatively reflect on the reputation of the farm. There is a common need for a basic awareness of the economic and public health implications of brucellosis in Kuwait: Brucellosis in small ruminants is a significant burden on animal and human health. Small ruminant owners and the government have to work together to afford and promote control or eradication campaigns. Despite the potential zoonotic and economic impact of brucellosis, the epidemiologic situation of *Brucella* infection in Kuwait is not well investigated and further studies are required.

## 5. Conclusions

Brucellosis is prevalent nationwide in sheep herds of Kuwait. The current study is the only large-scale survey on brucellosis in sheep and sheep holders recently conducted in the five districts with significant animal production in Kuwait. The high herd seroprevalence of 89% among tested herds is alarming, and control strategies have to continue accordingly. The test-and-slaughter strategy has begun and mass vaccination should be implemented promptly. Isolation of *B. melitensis*, the *Brucella* species with the highest impact on human health from milk and tissue samples highlights the continuous and now emerging significant risk for humans. This risk is already mirrored by the health status of the sheep holders and farmworkers with more than half of the herd owners (56.6%) seropositive for *Brucella*.

## Figures and Tables

**Table 1:** Number of sheep herds and animals sampled for investigation of brucellosis in Kuwait between 2016 and 2019.

Area	Number of Herds	Number of Animals	Status of Herd	Year
Al-Abdali	60	12,515	Apparent healthy, non-vaccinated	2017–2019
Al-Wafrah	45	26,225	Apparent healthy, non-vaccinated	2016–2017
Kabd	41	8290	Apparent healthy, non-vaccinated	2017–2019
Al-Salmi	34	8876	Apparent healthy, non-vaccinated	2017–2019
Al-Jahra	23	6715	Apparent healthy, non-vaccinated	2016–2017
Al-Jahra	12	2253	Vaccinated, *B. melitensis* Rev.1	2018
Al-Wafrah	8	2180	Vaccinated, *B. melitensis* Rev.1	2018
Total	223	67,054		

**Table 2:** Herd prevalence of sheep brucellosis in Kuwait between 2016 and 2019 showing numbers and percentages of positive herds and animals in five selected districts.

Districts	No. of Tested Herds (Animals)	No. of Positive Herds (%)	% of Positive Animals (%)
Al-Abdali	60 (12,515)	52 (86.7%)	1115 (9%)
Kabd	41 (8290)	31 (75.6%)	392 (4.7%)
Al-Salmi	34 (8876)	30 (88.24%)	511 (5.8%)
Al-Jahra	23 (6715)	23 (100%)	725 (10.8%)
Al-Wafra	45 (26,225)	45 (100%)	1720 (6.5%)
Total non-vaccinated herds	203 (62,621)	181 (89%)	4463 (7%)
Al-Jahra, vaccinated herds	12 (2253)	4 (33.3%)	97 (4.3%)
Al-Wafra, vaccinated herds	8 (2180)	2 (25%)	112 (5%)
Total vaccinated herds	20 (4433)	6 (30%)	209 (4.7%)

**Table 3:** Results for *Brucella* isolation from milk and tissue samples of seropositive cases and aborted fetuses.

Animals	Aborted Foeti		Milk Samples		Tissue Specimens		Total Examined Samples	Total Positive Culture (%)
	No. of Samples	No. of Isolates (%)	No. of Samples	No. of Isolates (%)	No. of Samples	No. of Isolates (%)		
Culture positive	8	8(100%)	30	10(33.3%)	8	2(25%)	46	20(43.5%)

**Table 4:** Numbers of seropositive human cases among sheep holders correlating to the number of positive sheep in each herd.

Herd ID	No. of Sheep within Herd	No. of Positive Sheep	No. of Tested Sheep Holders	No. of Positive Sheep Holders (%)	Region
1	120	10	4	2 (50%)	Al-Wafra
2	70	15	2	1 (50%)	Al-Wafra
3	300	32	5	2 (40%)	Al-Wafra
4	500	65	5	2 (40%)	Al-Salmi
5	150	33	3	3 (100%)	Al-Salmi
6	2700	130	8	6 (75%)	Al-Jahra
7	170	40	3	1 (33.3%)	Al-Jahra
Total	4010	325	30	17 (56.6%)	
